# Educational video-assisted versus conventional informed consent for trauma-related debridement surgery: a parallel group randomized controlled trial

**DOI:** 10.1186/s12910-018-0264-7

**Published:** 2018-03-09

**Authors:** Yen-Ko Lin, Chao-Wen Chen, Wei-Che Lee, Yuan-Chia Cheng, Tsung-Ying Lin, Chia-Ju Lin, Leiyu Shi, Yin-Chun Tien, Liang-Chi Kuo

**Affiliations:** 1 Division of Trauma and Surgical Critical Care, Department of Surgery, Kaohsiung Medical University Hospital, Kaohsiung Medical University, Kaohsiung, Taiwan; 2 Department of Medical Humanities and Education, College of Medicine, Kaohsiung Medical University, Kaohsiung, Taiwan; 3 Department of Emergency Medicine, College of Medicine, Kaohsiung Medical University, Kaohsiung, Taiwan; 4 College of Nursing, Kaohsiung Medical University, Kaohsiung, 807 Taiwan; 5 Department of Health Policy and Management, Bloomberg School of Public Health, Johns Hopkins University, Baltimore, MD USA; 6 Department of Orthopedics, Kaohsiung Medical University Hospital, Kaohsiung Medical University, Kaohsiung, Taiwan; 7 Department of Orthopedics, College of Medicine, Kaohsiung Medical University, Kaohsiung, Taiwan

**Keywords:** Informed consent, Trauma, Patient knowledge, Patient satisfaction, Emergency department

## Abstract

**Background:**

We investigated whether, in the emergency department (ED), educational video-assisted informed consent is superior to the conventional consent process, to inform trauma patients undergoing surgery about the procedure, benefits, risks, alternatives, and postoperative care.

**Methods:**

We conducted a prospective randomized controlled trial, with superiority study design. All trauma patients scheduled to receive trauma-related debridement surgery in the ED of Kaohsiung Medical University Hospital were included. Patients were assigned to one of two education protocols. Participants in the intervention group watched an educational video illustrating informed consent information, whereas those in the control group read an informed consent document. The primary outcome was knowledge scores and the secondary outcome was assessment of patient satisfaction. A multivariable regression model, with predefined covariates, was used to analyze differences in knowledge scores and patient satisfaction levels between the groups.

**Results:**

A total of 142 patients were enrolled, with 70 and 72 assigned to the intervention and control groups, respectively. Mean knowledge scores were higher in the intervention (72.57 ± 16.21 (SD)) than in the control (61.67 ± 18.39) group. By multivariate analysis, the intervention group had significantly greater differences in knowledge scores (coefficient: 7.646, 95% CI: 3.381–11.911). Age, injury severity score, and baseline knowledge score significantly affected the differences in knowledge scores. Significant improvements were observed in patients’ perception of statements addressing comprehension of the information provided, helpfulness of the supplied information for decision making, and satisfaction with the informed consent process. Multivariate analysis showed significant correlations between video education and patient satisfaction.

**Conclusions:**

Both the educational approach and severity of injury may have an impact on patient understanding during the informed consent process in an emergency environment. Video-assisted informed consent may improve the understanding of surgery and satisfaction with the informed consent process for trauma patients in the ED. Institutions should develop structured methods and other strategies to better inform trauma patients, facilitate treatment decisions, and improve patient satisfaction.

**Trial registration:**

The ClinicalTrials.gov Identifier is NCT01338480. The date of registration was April 18, 2011 (retrospectively registered).

**Electronic supplementary material:**

The online version of this article (10.1186/s12910-018-0264-7) contains supplementary material, which is available to authorized users.

## Background

Informed consent is not simply a document but rather a process [1–6]. During the informed consent process, it is ethically essential and legally imperative for physicians to provide information concerning invasive procedures, which describes the risks, benefits, and alternatives [7–9]. It is crucial that patients have sufficient understanding of the process and associated risks prior to providing their consent for any medical procedure. Only once such information is understood can patients make suitable individual treatment choices [10–13].

Trauma is one of the leading causes of death and disability as well as one of the top causes of mortality in children and young adults. It is, therefore, a major public health problem worldwide [14]. Obtaining valid informed consent from trauma patients in the emergency department (ED) is a challenging and time-consuming process. Because of the involuntary nature of emergency care, informed consent is the only way to respect patient autonomy [15, 16]. As with most situations occurring in emergency settings, time constraint, stress, and distress caused by acute symptoms or pain often make it difficult for patients and their families to absorb and understand the pertinent information needed for them to provide valid consent [16–22].

In our clinical experience, investigators have found that trauma patients have difficulty retaining the vast amount of information presented to them. In addition, during the traditional consent process, patients often cannot visualize how the surgery will take place. Furthermore, informed consent is usually obtained by residents or chief residents for most surgeries or procedures, especially in the ED. Residents may not have enough clinical experience to anticipate many unforeseen risks and complications of treatment. Moreover, some residents may not possess the communication skills required to explain the information in detail. These issues result in incomplete information being delivered to patients. Therefore, a cooperative effort by health care providers should be focused on developing more effective ways to convey information that will help patients and family members to make rational decisions, even under the most demanding conditions.

Availability of educational aids and supporting materials is important for the informed consent process; however, the manner in which this information is delivered is also important [23]. Traditional delivery of treatment information is usually via verbal and/or written communication. However, studies have showed that patients might have poor understanding of the information presented to them by these traditional methods [24–28]. Using an educational video to assist in preoperative discussion may increase both patient satisfaction and their understanding of the information [9, 29–33]. Several studies have showed that using videos to educate patients leads to higher patient satisfaction and enhanced patient knowledge concerning the procedures and their risks [34–36]. Although clinical studies in other medical areas have showed that video-informed patients retain more information, the use of video information among trauma patients in the ED has not been previously studied.

Our study investigated whether educational video-assisted informed consent was superior to the conventional consent process for informing trauma patients before undergoing surgery in the ED.

## Methods

### Conceptual framework

**Fig. 1:**
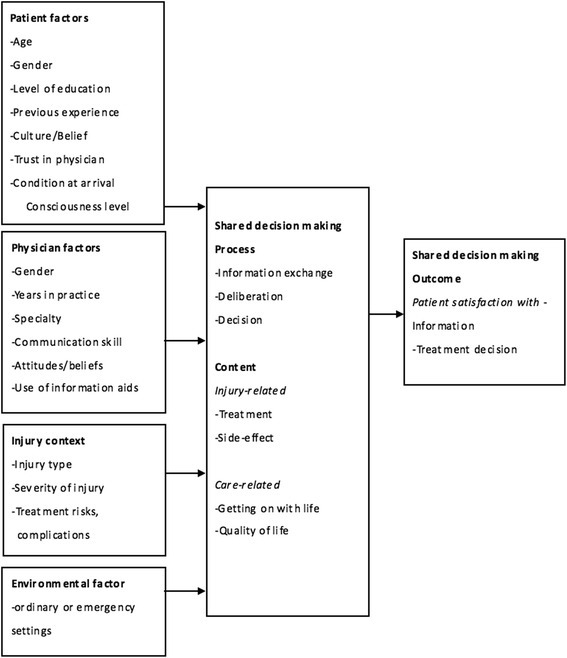
Conceptual framework

The conceptual framework of our work captured the complex realities of obtaining valid informed consent. The shared decision-making model developed by Leon-Carlyle et al. [37] for surgical consultation was modified and applied to the conceptual framework of this study. We measured a variety of parameters, including patient factors, physician factors, injury context, environmental factors affecting information exchange, as well as the patients’ deliberation and their willingness to voluntarily make treatment decisions and provide consent. We also assessed the impacts on patient satisfaction with the information and treatment decisions (Fig. 1).

### Intervention tool

Audiovisual videos and a knowledge measurement tool were designed and developed [38]. The modified Delphi technique was applied to reach a consensus among a panel of experts, leading to development of the video content and questions measuring the understanding of informed consent for specific surgeries among trauma patients. The video describing the surgery was developed using advanced two-dimensional (2-D) graphics technology. The final version of the video included seven sections: “Choosing the Appropriate Procedure”, “Medical History”, “Anesthesia”, “The Procedure, Benefits, and Risks”, “Alternatives”, “Postoperative Recovery” and “Wound Care”. The audio narrative described what was displayed in the video. Written subtitles and captions were also added.

A questionnaire to measure knowledge was also developed and tested. This measurement tool collected patient demographic data including age, sex, and level of education. Questions measuring patient knowledge about the informed consent were selected based on consensus of the experts and addressed the content of the video. The questions were written in a multiple-choice format, with 20 questions initially developed. These were distributed to the panel of experts and were also pilot tested to further refine the questionnaire. The final knowledge measurement questionnaire comprised 10 questions that were equally weighted in scoring (see Additional file 1: Appendix). The questionnaire also included three questions, answered using a five-point Likert scale, to assess satisfaction with the informed consent process.

### Participants

Adult trauma patients in the ED of Kaohsiung Medical University Hospital (KMUH) were included in the study. Those patients scheduled to receive surgical debridement for complicated injuries of the upper and lower extremities, under either general or epidural anesthesia, were eligible for enrollment provided that a trained research associate was available in the ED when the patient arrived. Facial wounds were excluded because of cosmetic concerns. Wounds involving tendon rupture, nerve injury, compound fracture, and vascular or crush injuries were also excluded because these required different postoperative rehabilitation programs. Patients who were clinically unstable, refused to participate, or were unable to speak and read Mandarin or comprehend the process of this study were also excluded. Because of the uncertainties associated with traumatic injury, eligible cases were missed if a research associate was not available. If an eligible patient was missed, the missed case and the reason it was missed were recorded in the study logbook. Research associates monitored surgical scheduling of the operating rooms using the hospital computer system to identify eligible cases. All research associates were specifically trained for their roles in the study.

### Study design

This study was a prospective randomized controlled trial with superiority design. Research associates approached eligible patients using a specific procedure, and then explained the study. If patients agreed to participate, written informed consent for the study was obtained. Patients who agreed to participate were randomly assigned to the video (intervention) group or the conventional informed consent (control) group. Group allocation was performed by simple randomization based on odd or even numbers generated using a computer-based random number generator; this procedure was conducted in a concealed manner. After randomization, participants were interviewed to collect their demographic information, including age, sex, and education level. Other variables, including injury severity score (ISS), whether they had been transferred, arrival time in the ED, and consulting physician, were collected from patient charts and the hospital computer system.

Participants in the control group received verbal information from health care providers and completed the knowledge measurement tool at baseline. Participants were then provided with an extended written consent form, which contained information about the surgery, to read and sign. The extended consent form included information similar to that on the video. This ensured that the same quality of information was delivered to all patients. Participants were also given an educational session with their health care provider to discuss concerns and ask questions. Finally, participants were asked to complete the knowledge measurement tool after the educational session. This included answering questions using a five-point Likert scale, to evaluate their satisfaction with the informed consent process.

Participants in the intervention group also received verbal information from health care providers and completed a knowledge measurement tool at baseline, before the intervention. These participants were then shown an educational video illustrating the procedure, risks, benefits, alternatives, and postoperative care relevant to the surgery. All participants watched the video on a single portable computer used for the study; the volume was adjusted to ensure that all could hear the narrative. A research associate provided assistance, as needed, while participants watched the video, to ensure that they completed the process and that their questions were addressed. Watching the video took approximately 15 min. If they had any further questions about the surgery, participants had the opportunity to speak with their health care provider after the video education session. This question and answer session created the same opportunity as that provided to the control group during the conventional informed consent process. After the question and answer session, patients in the intervention group completed the same knowledge test and satisfaction measurements as those in the control group.

In our ED, the senior and chief residents are the health care providers responsible for obtaining informed consent for the surgery. Residents obtaining informed consent were blinded to the knowledge measurement results, and the research associate who assessed the outcomes was blinded to the interventions.

### Data processing and statistical analysis

The primary outcome was determined by quantitative scores from 0% to 100%, representing patients’ understanding of the procedure, benefits, risks, alternatives, and postoperative care. Questions were in multiple-choice formats and quantitative scores on the written test were calculated. Secondary outcomes were evaluated using a five-point Likert ordinal satisfaction scale to represent patient satisfaction with the informed consent process. The frequency of refusal to provide consent was also recorded.

Data collected from participants were recorded by participant number, with no specific individual identifying information. This method was followed to protect patient privacy and ensure patient confidentiality. Collected data included patient demographics, ISS, whether the patient was transferred from another hospital, arrival time in the ED, and the names of treating physicians. Descriptive statistics were used to analyze the baseline characteristics of the control and intervention groups. Mean and standard deviations (SD) were calculated for continuous variables, if normally distributed, and proportions were calculated for categorical variables. Fisher’s exact test was performed for binary, ordinal, and categorical variables. Mean scores on the knowledge measurement tool before and after the educational sessions were compared using the Student’s *t-*test between each group and the paired *t-*test within each group. Differences in knowledge scores were calculated by subtracting values obtained before the education session from those after the education session. Independent factors found to be associated with the differences in knowledge scores and patient satisfaction, by univariate analysis, or being clinically important, were subsequently entered into multivariable regression models. A multiple linear regression model, a linear regression model with backward elimination, and a multiple ordinal logistic regression model for the differences in knowledge scores with predefined covariates was used. A multivariable logistic regression model of patient satisfaction, with predefined covariates, was applied as were likelihood ratio tests for the multivariable models. A threshold of *p* values less than 0.05 was determined for variables as to whether they were considered to be significant, and 95% CI were computed. All data analyses were conducted using Stata version 10.0 (StataCorp LP, College Station, TX, USA).

## Results

**Fig. 2:**
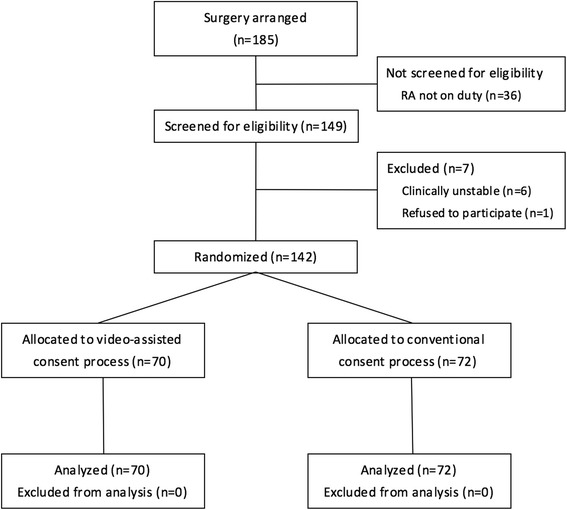
Profile of randomized controlled trial. (RA, Research associate)

A total of 185 adult patients were scheduled to receive surgical debridement during the study period (Fig. 2). A research associate was available to enroll 149 of the 185 patients. Of these, one declined to participate because they reported being “too nervous” and six were excluded because they were clinically unstable. Data were thus obtained for 142 subjects, as summarized in Table 1. There were 72 participants in the control group and 70 in the intervention group. There were no notable differences in baseline characteristics between the control and intervention groups. No adverse or unintended effects were noted in either group.

**Table 1:** Baseline characteristics

Characteristics	Control (*n* = 72)	Intervention (*n* = 70)
Age (years)	Number (%)	Number (%)
< 20	2 (2.8)	9 (12.9)
20–29	23 (31.9)	25 (35.7)
30–39	17 (23.6)	9 (12.9)
40–49	12 (16.7)	12 (17.1)
50–59	13 (18.1)	10 (14.3)
60–69	2 (2.8)	3 (4.3)
> 69	3 (4.2)	2 (2.9)
Male	43 (59.7)	36 (51.4)
Education		
< High school	13 (18.1)	8 (11.4)
High school	26 (36.1)	27 (38.6)
College	33 (45.8)	35 (50.0)
Injury severity score > 4	17 (23.6)	13 (18.6)
Transferred	16 (22.2)	19 (27.1)
Arrival time, 8–16 h	30 (41.7)	36 (51.4)
Physician		
Physician A	18 (25.0)	16 (22.9)
Physician B	7 (9.7)	10 (14.3)
Physician C	14 (19.4)	6 (8.6)
Physician D	11 (15.3)	12 (17.1)
Physician E	9 (12.5)	14 (20.0)
Physician F	13 (18.1)	12 (17.1)

### Knowledge measurements

**Fig. 3:**
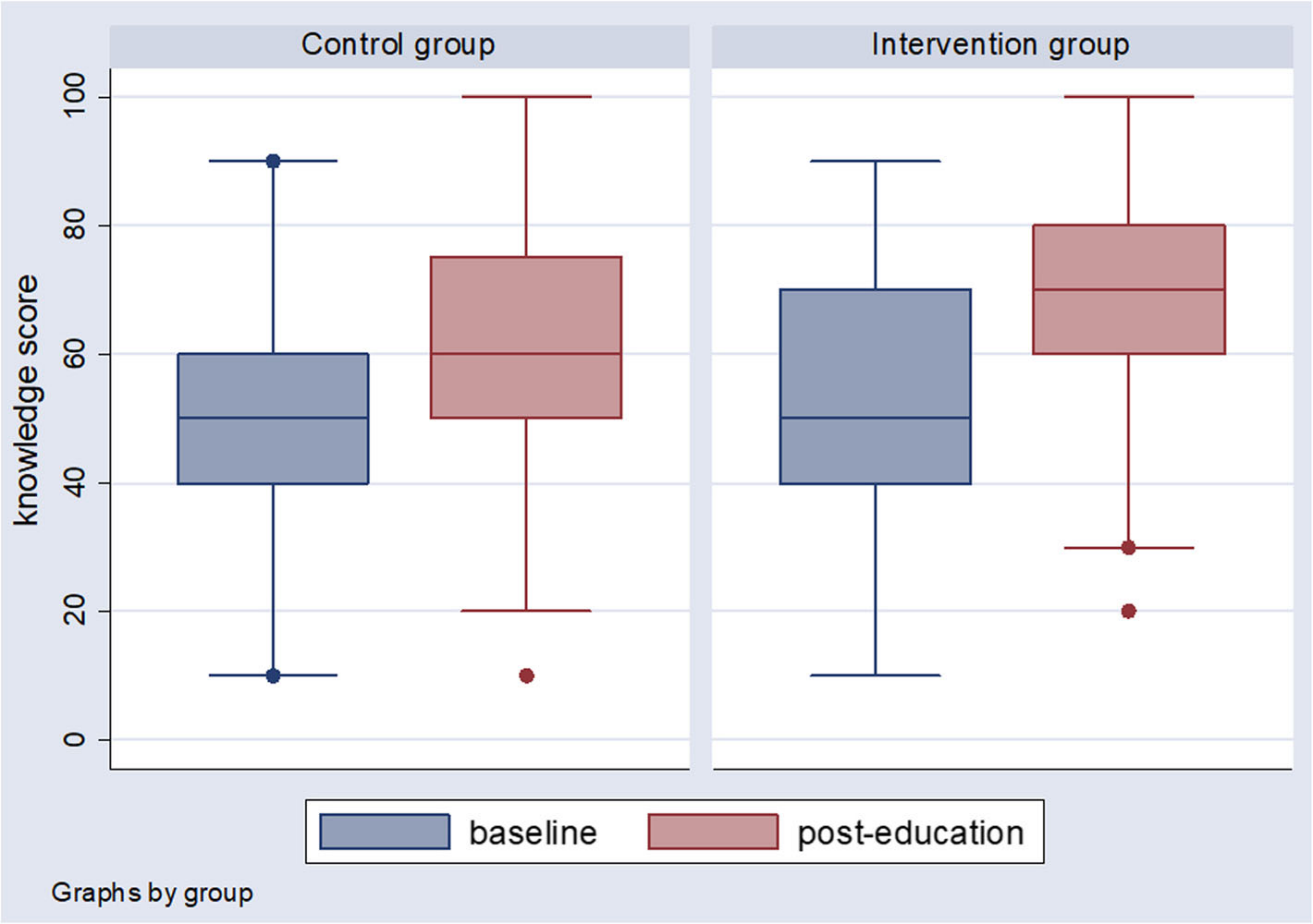
Baseline and post-education knowledge scores in control and intervention groups in boxplots. The line in the box means the median. The lower line of the box means the lower quartile, and the upper line of the box means the upper quartile

Table 2 and Fig. 3 summarize the major outcomes for all study participants. Individual performances on the knowledge test showed that patients in the two groups had no significant differences in baseline knowledge scores, and participants in the intervention group had greater understanding after the educational session compared with those in the control group (mean knowledge scores 72.57 and 61.67, respectively). Participants in both groups had higher knowledge scores after education than at baseline. There were significant differences in knowledge scores after education, and the intervention group showed a greater improvement in knowledge scores after the educational session (mean difference of knowledge scores 18.71) than the control group (mean difference of knowledge scores 10.83). We also categorized the differences in knowledge score into three categories of “≦10”, “20”, and “≥30”. There were significant differences between two groups (Table 2).

**Table 2:** Baseline and post-education knowledge scores

Knowledge score	Control (*n* = 72)		Intervention (*n* = 70)		*p*-value^d^
	Mean	Standard Deviation	Mean	Standard Deviation	
Baseline	50.83	18.67	53.86	16.44	0.308
Post-education	61.67	18.39	72.57	16.21	< 0.001
^c^Difference	10.83	11.23	18.71	16.76	0.001^a^
	Number (%)		Number (%)		
≦10	49 (68.06)		31 (44.29)		0.001^b^
20	16 (22.22)		15 (21.43)		
≥ 30	7 (9.72)		24 (34.29)		

^a^Unequal variance test

^b^Fisher’s exact test

^c^Difference = (Post-education knowledge score) - (Baseline knowledge score)

^d^Intervention vs Control

Table 3 provides further detail for the comparison of subgroup baseline knowledge scores in the two groups. There were no significant differences between the control and intervention groups. Table 4 shows post-education knowledge scores in the different subgroups. The post-education knowledge scores were significantly greater in several subgroups than in the others. These included participants aged < 36 years, males, and those with ISS ≤ 4 in the intervention group. No matter the level of education (<High school or ≥High school), being transferred or not, and the arrival time (8–16 h or others), patients had significantly higher post-video educational scores in the intervention group. The consulting physician had no effect on the post-educational scores in either group.

**Table 3:** Baseline knowledge scores compared between control and intervention subgroups

Variable	Control			Intervention			*p*-value
	*n*	Mean	Standard deviation	*n*	Mean	Standard deviation	
Age (years)							
< 36	32	55.94	17.01	39	56.41	17.09	0.908
≥ 36	40	46.75	19.13	31	50.65	15.26	0.357
Sex							
Female	29	56.90	16.93	34	56.47	17.73	0.923
Male	43	46.74	18.86	36	51.39	14.96	0.236
Education							
< High school	13	35.39	19.42	8	46.25	9.16	0.157
≥ High school	59	54.24	16.84	62	54.84	16.96	0.845
Injury severity score							
≦4	55	51.09	18.82	57	54.04	16.89	0.385
> 4	17	50.00	18.71	13	53.08	14.94	0.631
Transferred							
Yes	16	52.50	20.49	19	55.26	14.67	0.646
No	56	50.36	18.29	51	53.33	17.17	0.389
Arrival time							
8–16 h	30	49.00	16.47	36	51.67	16.30	0.513
Others	42	52.14	20.19	34	56.18	16.52	0.351
Physician							
Physician A	18	56.67	16.80	16	52.50	14.38	0.446
Physician B	7	47.14	19.76	10	56.00	18.97	0.366
Physician C	14	47.86	19.29	6	53.33	21.60	0.581
Physician D	11	45.46	22.07	12	60.00	15.37	0.079
Physician E	9	52.22	17.87	14	50.00	20.00	0.789
Physician F	13	51.54	18.64	12	52.50	11.38	0.879

**Table 4:** Post-education knowledge scores compared between control and intervention subgroups

Variable	Control			Intervention			*p*-value
	*n*	Mean	Standard deviation	*n*	Mean	Standard deviation	
Age (years)							
< 36	32	65.31	17.22	39	78.21	13.93	< 0.001
≥ 36	40	58.75	18.97	31	65.48	16.30	0.120
Sex							
Female	29	66.21	12.93	34	71.47	17.43	0.185
Male	43	58.61	20.88	36	73.61	15.15	< 0.001
Education							
< High school	13	44.62	18.98	8	66.25	15.06	0.013
≥ High school	59	65.42	16.12	62	73.39	16.29	0.008
Injury severity score							
≦4	55	62.91	18.43	57	74.39	16.48	< 0.001
> 4	17	57.65	18.21	13	64.62	12.66	0.249
Transferred							
Yes	16	58.75	17.84	19	72.11	14.75	0.021
No	56	62.50	18.61	51	72.75	16.86	0.004
Arrival time							
8–16 h	30	61.00	16.47	36	72.22	18.07	0.011
Others	42	62.14	19.82	34	72.94	14.26	0.009
Physician							
Physician A	18	65.00	14.65	16	71.13	20.24	0.186
Physician B	7	58.57	24.78	10	73.00	13.37	0.140
Physician C	14	57.86	18.05	6	70.00	10.95	0.146
Physician D	11	60.00	23.24	12	74.17	13.79	0.087
Physician E	9	67.78	14.81	14	72.14	18.88	0.564
Physician F	13	60.00	19.15	12	71.67	16.42	0.117

Table 5 shows the differences in knowledge scores among the subgroups. Differences in knowledge scores were significantly greater in the intervention versus control group, in certain subgroups (age < 36 years, male, education level above high school, ISS ≤ 4, and physician F). The educational video may have had a greater impact on participants in those subgroups because they had greater differences between baseline and post-education knowledge scores. Patients with less than a high school-level education showed a trend toward higher differences in knowledge scores in the intervention group, although these were not statistically significant. No matter being transferred or not, and the arrival time (8–16 h or others), patients had significantly greater differences in knowledge score in the intervention group. The difference of knowledge scores between intervention and control group for age < 36 and age ≥ 36 reached statistical significance (interaction *p*-value was 0.046).

**Table 5:** The difference of knowledge scores compared between control and intervention subgroups

Analysis variable	Control			Intervention			*p*-value	*p*-value for interaction of experimental group and analysis variable
	*n*	Mean	Standard deviation	*n*	Mean	Standard deviation		
Age (years)								
< 36	32	9.38	11.34	39	21.80	17.30	< 0.001^a^	0.046
≥ 36	40	12.00	11.14	31	14.84	15.46	0.392^a^	
Sex								
Female	29	9.31	8.84	34	15.00	15.42	0.084^a^	0.329
Male	43	11.86	12.58	36	22.22	17.42	0.003^a^	
Education								
< High school	13	9.23	11.15	8	20.00	15.12	0.073^a^	0.624
≥ High school	59	11.19	11.31	62	18.55	17.07	0.004^a^	
Injury severity score								
≦4	55	11.82	11.56	57	20.35	17.11	0.003^a^	0.427
> 4	17	7.65	9.70	13	11.54	13.45	0.388^a^	
Transferred								
Yes	16	6.25	9.57	19	16.84	14.55	0.015^a^	0.550
No	56	12.14	11.40	51	19.41	17.60	0.014^a^	
Arrival time								
8–16 h	30	12.00	12.70	36	20.56	18.66	0.031^a^	0.711
Others	42	10.00	10.12	34	16.76	14.51	0.025^a^	
Physician								
Physician A	18	8.33	7.86	16	17.50	17.32	0.066^a^	0.970
Physician B	7	11.43	9.00	10	17.00	22.63	0.495^a^	
Physician C	14	10.00	10.38	6	16.67	22.51	0.514^a^	
Physician D	11	14.55	15.08	12	18.33	17.49	0.583^a^	
Physician E	9	15.56	17.40	14	22.14	14.24	0.358^a^	
Physician F	13	8.46	8.01	12	19.17	11.65	0.015^a^	

^a^Unequal variance test

Table 6 shows the results of differences in knowledge scores by simple regression models, multiple linear regression model, and linear regression model with backward elimination as well as multiple ordinal logistic regression model. A multiple linear regression model was applied to study the adjusted impact of video education, controlling for predefined covariates. The results showed that video education significantly increased the differences in knowledge scores. The average difference in knowledge score was increased by 7.646 points. Moreover, age, ISS, and baseline knowledge score also significantly affected the differences in knowledge scores, with coefficients of − 0.161, − 0.842 and − 0.379, respectively. Lower age, lower ISS, and lower baseline knowledge scores were all correlated with higher differences in knowledge scores. The coefficients of these variables were similar with those in the simple linear regression model. The coefficients remained similar in the linear regression model with backward elimination. In the multiple ordinal logistic regression model, the adjusted odds ratio for the intervention group suggests that the intervention increased the differences in knowledge score. Adjusted odds ratio for the difference in knowledge scores in the intervention group was 4.724 (95% CI: 2.140–10.428).

**Table 6:** Differences in knowledge scores by regression models

	Simple linear regression model		Multiple linear regression model		Linear regression model with backward elimination		Multiple ordinal logistic regression model	
	Coefficient	95% CI	Coefficient	95% CI	Coefficient	95% CI	Odds ratio	95% CI
Intervention group	7.881***	3.160–12.602	7.646***	3.381–11.911	7.602***	3.428–11.776	4.724***	2.140–10.428
Age	−0.099	−0.264-0.065	− 0.161*	−0.318--0.004	− 0.189**	− 0.333--0.046	0.978	0.950–1.007
Sex (reference group = female)	4.201	−0.681-9.083	1.420	−2.943-5.784			1.543	0.719–3.310
Education (reference group = <high school)	1.625	−5.272-8.523	4.021	−2.577-10.619			1.428	0.443–4.600
Injury severity score	−1.181***	−1.889--0.472	−0.842*	−1.513--0.171	−0.923**	− 1.54--0.300	0.771**	0.645–0.921
Transferred (reference group = non-transferred)	−3.607	−9.261-2.046	−1.772	−6.724-3.181			0.633	0.258–1.549
Arrival time (reference group = other)	3.640	−1.235-8.516	0.775	−3.730-5.280			1.355	0.633–2.903
Physician	1.357	−5.072-7.787	−1.307	−7.200-4.587			1.107	0.405–3.030
Baseline knowledge score	−0.319***	−0.44--0.190**	−0.379***	− 0.508--0.249	−0.373***	− 0.494--0.252	0.946***	0.922–0.971
Constant			36.410***	22.119–50.701	40.522***	30.693–50.351		
			R squared = 0.329		R squared = 0.316		Likelihood ratio test for model: *χ2* = 54.18; *p* < 0.0001	

*p < 0.05; **p < 0.01; ***p < 0.001. Sample size of regression model = 142

### Patient satisfaction

Patient satisfaction data, as measured on a five-point scale, are summarized in Table 7. There were significant differences between the control and intervention groups in the responses to the following statements: “I can comprehend the information that health care providers supplied for the surgery.”; “The information that health care providers supplied will help me make decisions for the surgery.”; and “I am satisfied with the informed consent process for the surgery.” No patient in either group refused to provide their consent for surgery. Additional file 2: Table S1 shows a comparison of the results of subgroup analysis for patient satisfaction between the control and intervention groups, for responses to the above three statements. To summarize patient satisfaction, we further categorized the five-point Likert scale into two categories of “Strongly agree” and “Other”.

**Table 7:** Comparison of satisfaction between control and intervention groups

Outcome	Control number (%)	Intervention number (%)	*p*-value
I can comprehend the information that health care providers supplied for the surgery			< 0.001^b^
Strongly agree	22 (30.6)	43 (61.4)	
Agree	42 (58.3)	27 (38.6)	
Fair	7 (9.7)	0 (0.0)	
Disagree	0 (0.0)	0 (0.0)	
Strongly disagree	1 (1.4)	0 (0.0)	
The information that the health care providers supplied will help me make decisions for the surgery			< 0.001^b^
Strongly agree	29 (40.3)	49 (70.0)	
Agree	38 (52.8)	21 (30.0)	
Fair	4 (5.6)	0 (0.0)	
Disagree	0 (0.0)	0 (0.0)	
Strongly disagree	1 (1.4)	0 (0.0)	
I am satisfied with the informed consent process for the surgery			< 0.001^b^
Strongly agree	31 (43.1)	52 (74.3)	
Agree	37 (51.4)	18 (25.7)	
Fair	4 (5.6)	0 (0.0)	
Disagree	0 (0.0)	0 (0.0)	
Strongly disagree	0 (0.0)	0 (0.0)	

^b^Fisher’s exact test

For the statement “I can comprehend the information that health care providers supplied for the surgery”, participants in certain subgroups (age < 36 years, education level high school and above, ISS < 4, and physician A) responded “Strongly agree” considerably more in the intervention group than in the control group. For other parameters (sex, transfer, arrival time, and baseline knowledge scores), all patients had considerably more responses of “Strongly agree” in the intervention versus control group, regardless of subgroup. Patients with higher differences in knowledge scores had considerably more “Strongly agree” ratings in the intervention group than in the control group.

For the statement “The information that health care providers supplied will help me make decisions for the surgery”, patients in certain subgroups (less than high school-level education, ISS > 4, being transferred, and not treated by physician A) showed the same percentage of “Strongly agree” ratings in both the intervention and control groups. Participants in other subgroups responded with “Strongly agree” more often in the intervention than in the control group. Patients with higher differences of knowledge scores also responded with “Strongly agree” considerably more in the intervention than in the control group.

For the statement “I am satisfied with the informed consent process for the surgery”, patients in certain subgroups (age ≥ 36 years, females, less than high school-level education, ISS > 4, and not treated by physician A) gave a rating of “Strongly agree” in both the intervention and control groups. Patients in other subgroups responded with “Strongly agree” more often in the intervention than in the control group. Patients with higher differences in knowledge scores gave a rating of “Strongly agree” more often in the intervention group than in the control group.

Multivariable logistic regression models of patient satisfaction, controlling for predefined covariates, are shown in Table 8. The adjusted odds ratio for the intervention group suggests that the intervention improved patients’ perception of satisfaction. The adjusted odds ratio for the statements, “I can comprehend the information that health care providers supplied for the surgery”, “The information that health care providers supplied can help me make decisions for the surgery”, and “I am satisfied with the informed consent process for the surgery” were 3.299 (95% confidence interval [CI]: 1.614–6.746), 3.246 (95% CI: 1.567–6.727), and 3.702 (95% CI: 1.747–7.843), respectively.

**Table 8:** Multivariable logistic regression model for satisfaction

	I can comprehend the information that the health care providers supplied for the surgery		The information that the health care providers supplied will help me make decisions for the surgery		I am satisfied with the informed consent process for the surgery	
	Odds ratio	95% CI	Odds ratio	95% CI	Odds ratio	95% CI
Intervention group (reference group = control group)	3.299***	1.614–6.746	3.246**	1.567–6.727	3.702***	1.747–7.843
Age (reference group = age < 36)	0.703	0.326–1.515	0.379*	0.175–0.822	0.371*	0.168–0.821
Sex (reference group = female)	0.552	0.260–1.175	0.770	0.359–1.653	0.578	0.264–1.264
Education (reference group = <high school)	1.243	0.412–3.752	1.230	0.414–3.654	1.119	0.379–3.303
Injury severity score (ISS) (reference group = ISS≦4)	0.654	0.268–1.595	0.849	0.353–2.039	1.031	0.421–2.523
Transferred (reference group = non-transferred)	1.307	0.578–2.955	0.883	0.383–2.036	0.669	0.287–1.563
Arrived time (reference group = other)	1.222	0.582–2.568	0.972	0.459–2.058	0.923	0.430–1.981
Physician (reference group = physician A)	0.733	0.313–1.715	0.904	0.381–2.145	0.938	0.387–2.273
Baseline knowledge score (BKS) (reference group = BKS < 60)	0.965	0.453–2.057	1.195	0.557–2.567	1.134	0.520–2.473
Likelihood ratio test for model	*χ2* = 19.41; *P* = 0.022		*χ2* = 22.13; *P =* 0.009		*χ2* = 24.83; *P* = 0.003	

*p < 0.05; **p < 0.01; ***p < 0.001. Sample size of regression model = 142

## Discussion

The results of our study represent some conceptual advances over previously published work. Our findings indicate that both different informational aids and severity of injury may impact patient understanding during the informed consent process in an emergency environment. Though a difference in knowledge score represents an average of less than a one-question difference between two groups, our results showed a significant trend where trauma patients had a better understanding of information provided by the educational video compared with that obtained from the traditional informed consent process. This may recall the theory of multimedia learning called the multimedia principle, proposed by Mayer, which states that “people learn more deeply from words and pictures than from words alone” [39]. In our sample, patients showed greater satisfaction with the informed consent process in the intervention group than in the control group. To our knowledge, ours is the first study to use educational videos to improve the informed consent process for trauma patients in the ED.

Although informed consent is an essential issue for physicians, it has been reported that “most physicians do not devote appropriate importance to it in their daily duties” [40]. Similar concerns have been reported in Japan. Some physicians might simply try to obtain a consent signature without a deep understanding of the fundamental ethical principles of informed consent [41]. One study in South Africa reported that doctors might understand the general concept and have knowledge of the informed consent process, but its practice remains inadequate [42]. Furthermore, one study showed that the administration and documentation of informed consent for surgical health care at university teaching hospitals was inadequate [43]. Hence, patients’ needs may not be adequately met by the current principles for consent to treatment, particularly in emergencies.

Most patients would like to have more information when making medical decisions, regardless of their level of education [44]. Patient factors (including age, education level, previous experience with surgery, and consciousness level), physician factors (including years in practice, communication skills, and use of informational aids), and injury context (including injury type and severity) may affect information exchange. Other factors that have an impact on the process are the patient’s deliberation and whether the patient voluntarily agrees to make treatment decisions or to provide consent [37, 44]. In our study, we found that younger age, injury severity, baseline knowledge score, and use of an educational video were significant factors predicting increased patient knowledge and understanding. We observed a higher impact of video education on understanding among younger patients. Different patient populations might have different preferences for the type of informational aid used. A tailor-made informational aid might be necessary for patients to improve understanding and satisfaction. Furthermore, the severity of injury has an impact on a patient’s understanding. A previous study indicated that patients with acute medical conditions had poor understanding of study goals, risks, and benefits [45]. In our study, patients with more severe injuries had lower post-education knowledge scores and relatively limited improvement in knowledge after education. This finding may relate to the intrinsic difficulties of obtaining valid informed consent from trauma patients. Acute physical pain and emotional stress limit the ability of trauma patients to absorb the complex information needed to provide valid informed consent. These patients may have limited competence to be able to provide valid consent, and they may also have limited interest in participating in consent negotiations given their acute state. All these factors may undermine the validity of provided consent regardless of the conventional consent procedure or the benefits of using video as part of the consent procedure.

In our study, no patient in either group refused to provide their consent for surgery. The understanding of potential alternatives to debridement surgery is essential, as is understanding of the right to refuse surgery. It is reasonable to expect that patients in the ED will be strongly biased towards receiving the surgery suggested by health care professionals. Protection of autonomy through informed consent therefore requires significant effort to ensure that the patient understands the alternatives and their right to refuse an intervention.

In this study, the educational video increased post-education knowledge scores and the differences in knowledge scores, as well as patient satisfaction. Nehme et al. studied the effect of using multimedia consent programs for surgical procedures. They reported that it was difficult to conclude whether higher patient satisfaction was correlated with improved understanding or merely with the use of a multimedia program. [46] In our study, improved knowledge scores were associated with higher patient satisfaction. These results indicate that using the educational video itself improved both patient satisfaction and patient knowledge.

Time may influence the outcome of patient deliberations [47]. Theoretically, if patients have more time to study the provided information and deliberate, they will have better understanding. Fink et al. suggested that providing adequate time and using adjuncts for informed consent discussions may improve patient understanding [47]. In our study, we did not evaluate the time spent with each participant. However, we believe that the time required for participants to complete the consent process was similar in the intervention and control groups. We provided a similar amount of time for participants to read the written information as to watch the video and provided similar time for all participants to ask questions. Therefore, time might not have influenced the results of our study.

Some authors have reported that patients with lower educational levels may have improved understanding with additional interventions [48]. In our study, patients with lower education levels showed a trend, compared with more highly educated patients, toward greater differences of knowledge scores in the intervention than in the control group. However, this education level-associated effect was not significant. Moreover, higher patient satisfaction was not evident among patients with lower education levels. There are several possible reasons for these results. The sample sizes of the lower-education-level subgroups were small in our study so the differences may be inconclusive. In addition, the video design might not have been well suited to those patients. The video layout and narrative expression might influence patient understanding and satisfaction. Further research is needed to explore such associations.

In addition to its content, we believe that the quality of a video production can influence patient understanding and satisfaction, although theoretical and empirical studies have not supported this. We believe if a video is produced more attractively, its effectiveness in education might be greater. Actor roleplay, 2-D or 3-D graphics, or interactive computer programs could be used, perhaps with varying effects on patient outcome. Moreover, we believe that audio narratives can also influence the absorption of information by patients. A female voice may sound softer and a male voice may sound more authoritative. With different audio, the effectiveness of information delivery might vary. Further research is also needed to explore such associations and effects.

Video does not allow for immediate answers to patients’ questions. In addition, there may not be an opportunity for patients to repeat or focus on specific areas of concern in the video. An interactive program with a custom design would be ideal for addressing these limitations. However, it must be emphasized that such information aids should not replace the entire process of informed consent. Patients should have a chance to communicate with their health care providers. Informed consent is a crucial process in which patients and health care providers should have an opportunity to express their own opinions and values, exchange information, and gain mutual understanding.

Documentation is an important issue when informed consent is obtained electronically. It is necessary to consider how best to preserve consent documents, in accordance with institutional regulations and the law. Some have reported using electronic consent obtained by integrating electronic signatures into patient electronic records [49]; however, future study is needed to confirm its applicability and effectiveness.

Our study showed that watching the video improved knowledge and satisfaction among trauma patients during the informed consent process. However, it should be emphasized that major improvements are achieved when institutions devote effort to improving patient safety and quality of care, by conveying structured information and standardizing the process for trauma patients in the ED. We believe that improvements in patient outcomes have reflected such achievements. Institutions should make patient-centered health care a top priority, while also emphasizing improved quality of care in the ED [50]. In addition, ED staff must frequently communicate this principle to other health care personnel so as to provide appropriate care for trauma patients throughout their treatment.

This study has several strengths. The randomization may have balanced the contributions of patient background and knowledge of the surgery between the groups. Baseline knowledge measurements were formally performed, thus limiting potential bias and accurately reflecting improvement after the intervention. Moreover, the study included several important elements that were not previously investigated, including use of a video containing the informed consent information for trauma surgery and having a study population selected from an emergency setting.

This study also has limitations. Some studies have reported that the effects of formal education on patient knowledge and satisfaction are uncertain [51]. Although our study produced some promising results, it was based on one pilot study and sought, at an exploratory stage, to investigate a viable alternative to current practice. This study represents only one specific surgery conducted at one institution, so the results may not be generalized to other surgeries or institutions. Furthermore, we did not measure patient understanding of the possible functional outcomes of surgery, the broader risks of surgery including anesthesia procedures, or of any alternatives to surgery. Trauma patients may have radically different injuries, requiring very different specific approaches. This makes it difficult for a single video to address every possible aspect of a particular situation. We recommend further multicenter randomized controlled trials to decrease the risk of bias. The baseline knowledge measurement may reflect accurate improvement in knowledge; however, it may potentially prime participants to pay attention to specific details during the educational component. The effects of this are unknown. Our study also did not evaluate effects of the educational video on patient anxiety. We believe that levels of anxiety might be higher for trauma patients in the ED than in other settings and that the educational video might alleviate some of this anxiety; however, further research will be needed to confirm this. In our study, information retention was also not evaluated. The ability to retain information and understanding in the longer term is important. The information provided as part of the informed consent procedure is not only relevant for the consent to surgery, but also for the time of rehabilitation. The advantage of using video as part of the informed consent process would be further substantiated if it also increased understanding in the longer, post­operative term. Further research is recommended to explore the effectiveness of an educational video on information retention and satisfaction among trauma patients. Moreover, patient literacy was not assessed in our study. Some participants might not have adequately read and understood the written informed consent document. Therefore, further research is also recommended to explore this relationship. Furthermore, the video in this study was not available in versions using various native dialects. Videos in different languages should be prepared and evaluated.

## Conclusions

Different educational approaches and severity of injury may both impact patient understanding during the informed consent process in an emergency environment. Our results indicated that educational videos are useful tools for improving the informed consent process for surgery among trauma patients. Video-assisted informed consent may improve understanding of the surgery as well as satisfaction with the process of informed consent in these patients. Future studies are recommended to confirm these preliminary findings in patients with different injury types and varying levels of severity. For trauma patients, an audiovisual aid may increase understanding of the surgery, facilitate medical decision making, and improve satisfaction. Institutions should develop strategies and structured methods to better inform trauma patients in this manner. For health care providers, an educational video may be useful to improve communication with patients, thereby further facilitating treatment decisions.

## Additional files

Additional file 1: **Appendix.** Knowledge measurement questionnaire. (DOCX 15 kb)

Additional file 2: **Table S1.** Comparisons of satisfaction between control and intervention subgroups. (DOCX 29 kb)

## Abbreviations

ED: Emergency department
ISS: Injury severity score
KMUH: Kaohsiung Medical University Hospital
