# Supplementary material for: Educational video-assisted versus conventional informed consent for trauma-related debridement surgery: a parallel group randomized controlled trial
Source: BMC Med Ethics. 2018 Mar 9;19:23. doi: 10.1186/s12910-018-0264-7 (PMC5845218; doi:10.1186/s12910-018-0264-7)
Supplement: Supplementary file 1 — Appendix. Knowledge measurement questionnaire. (DOCX 15 kb) [file 12910_2018_264_MOESM1_ESM.docx]

Additional file 1

| Appendix. Knowledge measurement questionnaire | |  |
| --- | --- | --- |
| Questions | | |
| 1. The purpose of the debridement surgery is to (1) relieve pain, (2) examine the infective pathogens, (3) remove necrotic tissue and foreign bodies from the wound, or (4) all of the above. | | |
| 1. Which of the following is a risk for surgical debridement? (1) The vessels, tendons, or nerves might be injured, (2) bacteria from the skin might affect the deep tissue and cause infection, or (3) both of the above. | | |
| 1. Which of the following might increase the risks of the procedure and postoperative complications? (1) Using pain killers, (2) using steroids, or (3) using antibiotics. | | |
| 1. Which of the following conditions might increase the risks of the procedure and postoperative complications? (1) Imbibing alcohol, (2) smoking, (3) drinking coffee, or (4) chewing betel nuts. | |  |
| 1. The appearance of the wound should be observed postoperatively. Which of the following is normal? (1) Redness over or around the wound. (2) The yellowish or green discharge has a bad odor, or more discharge is noted from the wound. (3) The body temperature is 37°C. (4) The skin at the edge of the wound remains wet. | |  |
| 1. When after injury should ice packing over the wound be started? (1) 1–3 days, (2) 3–6 days, or (3) longer than 6 days. | |  |
| 1. How long should the ice packing be performed each time? (1) 1–5 minutes, (2) 10–15 minutes, or (3) 30–60 minutes. | | |
| 1. Which of the following is not the purpose of ice packing? (1) To stop the bleeding, (2) increase circulation, or (3) alleviate pain. | | |
| 1. When after injury should hot packing be applied? (1) 1st day, (2) 2nd day, or (3) 3rd day or later. | | |
| 1. If the gauze adheres to the wound, what can you do when changing the dressing? (1) Remove it directly, (2) use hydrogen dioxide to rinse the gauze, or (3) use normal saline to rinse the gauze. | | |
