# Supplementary material for: Educational video-assisted versus conventional informed consent for trauma-related debridement surgery: a parallel group randomized controlled trial
Source: BMC Med Ethics. 2018 Mar 9;19:23. doi: 10.1186/s12910-018-0264-7 (PMC5845218; doi:10.1186/s12910-018-0264-7)
Supplement: Supplementary file 2 — Table S1. Comparisons of satisfaction between control and intervention subgroups. (DOCX 29 kb) [file 12910_2018_264_MOESM2_ESM.docx]

Additional file 2

Table S1 Comparisons of satisfaction between control and intervention subgroups

|  | I can comprehend the information that health care providers supplied for the surgery | | | | | The information that health care providers supplied will help me make decisions for the surgery | | | | | I am satisfied with the informed consent process for the surgery | | | | |
| --- | --- | --- | --- | --- | --- | --- | --- | --- | --- | --- | --- | --- | --- | --- | --- |
| Variable | Control | | Intervention | | *p*-value | Control | | Intervention | | *p*-value | Control | | Intervention | | *p*-value |
|  | Strongly agree, | Others | Strongly agree | Others |  | Strongly agree | Others | Strongly agree | Others |  | Strongly agree | Others | Strongly agree | Others |  |
| Age (years) |  |  |  |  |  |  |  |  |  |  |  |  |  |  |  |
| <36 | 10 (31.3) | 22 (68.7) | 26 (66.7) | 13 (33.3) | 0.004 | 17 (53.1) | 15 (46.9) | 31 (79.5) | 8 (20.5) | 0.023 | 16 (50.0) | 16 (50.0) | 34 (87.2) | 5 (12.8) | 0.001 |
| ≥36 | 12 (30.0) | 28 (70.0) | 17 (54.8) | 14 (45.2) | 0.051 | 12 (30.0) | 28 (70.0) | 18 (58.1) | 13 (41.9) | 0.029 | 15 (37.5) | 25 (62.5) | 18 (58.1) | 13 (41.9) | 0.099 |
| Sex |  |  |  |  |  |  |  |  |  |  |  |  |  |  |  |
| Female | 11 (37.9) | 18 (62.1) | 23 (67.7) | 11(32.3) | 0.024 | 13 (44.8) | 16 (55.2) | 24 (70.6) | 10 (29.4) | 0.045 | 15 (51.7) | 14 (48.3) | 26 (76.5) | 8 (23.5) | 0.063 |
| Male | 11 (25.6) | 32 (74.4) | 20 (55.6) | 16 (44.4) | 0.011 | 16 (37.2) | 27 (62.8) | 25 (69.4) | 11 (30.6) | 0.006 | 16 (37.2) | 27 (62.8) | 26 (72.2) | 10 (27.8) | 0.003 |
| Education |  |  |  |  |  |  |  |  |  |  |  |  |  |  |  |
| <High school | 4 (30.8) | 9 (69.2) | 3 (37.5) | 5 (62.5) | 1.000 | 4 (30.8) | 9 (69.2) | 4 (50.0) | 4 (50.0) | 0.646 | 5 (38.5) | 8 (61.5) | 4 (50.0) | 4 (50.0) | 0.673 |
| ≥High school | 18 (30.5) | 41 (69.5) | 40 (64.5) | 22 (35.5) | <0.001 | 25 (42.4) | 34 (57.6) | 45 (72.6) | 17 (27.4) | 0.001 | 26 (44.1) | 33 (55.9) | 48 (77.4) | 14 (22.6) | <0.001 |
| Injury severity score |  |  |  |  |  |  |  |  |  |  |  |  |  |  |  |
| ≦4 | 18 (67.3) | 37 (32.7) | 36 (63.2) | 21 (36.8) | 0.001 | 22 (40.0) | 33 (60.0) | 41 (71.9) | 16 (28.1) | 0.001 | 22 (40.0) | 33 (60.0) | 44 (77.2) | 13 (22.8) | <0.001 |
| >4 | 4 (23.5) | 13 (76.5) | 7 (53.9) | 6 (46.1) | 0.132 | 7 (41.2) | 10 (58.8) | 8 (61.5) | 5 (38.5) | 0.462 | 9 (52.9) | 9 (47.1) | 8 (61.5) | 5 (38.5) | 0.721 |
| Transferred |  |  |  |  |  |  |  |  |  |  |  |  |  |  |  |
| Yes | 4 (25.0) | 12 (75.0) | 14 (73.7) | 5 (26.3) | 0.007 | 6 (37.5) | 10 (62.5) | 13 (68.4) | 6 (31.6) | 0.095 | 5 (31.3) | 11 (68.7) | 14 (73.7) | 5 (26.3) | 0.018 |
| No | 18 (32.1) | 38 (67.9) | 29 (56.9) | 22 (43.1) | 0.012 | 23 (41.1) | 33 (58.9) | 36 (70.6) | 15 (29.4) | 0.003 | 26 (46.4) | 30 (53.6) | 38 (74.5) | 13 (25.5) | 0.003 |
| Arrival time |  |  |  |  |  |  |  |  |  |  |  |  |  |  |  |
| 8–16 h | 10 (33.3) | 20 (66.7) | 22 (61.1) | 14 (38.9) | 0.029 | 11 (36.7) | 19 (63.3) | 25 (69.4) | 11 (30.6) | 0.013 | 11 (36.7) | 19 (63.3) | 27 (75.0) | 9 (25.0) | 0.003 |
| Others | 12 (28.6) | 30 (71.4) | 21 (61.8) | 13 (38.2) | 0.005 | 18 (42.9) | 24 (57.1) | 24 (70.6) | 10 (29.4) | 0.021 | 20 (47.6) | 22 (52.4) | 25 (73.5) | 9 (26.5) | 0.034 |
| Physician |  |  |  |  |  |  |  |  |  |  |  |  |  |  |  |
| Physician A | 4 (22.2) | 14 (77.8) | 13 (81.3) | 3 (18.8) | 0.002 | 7 (38.9) | 11 (61.1) | 12 (75.0) | 4 (25.0) | 0.045 | 7 (38.9) | 11 (61.1) | 13 (81.3) | 3 (18.8) | 0.017 |
| Physician B | 2 (28.6) | 5 (71.4) | 6 (60.0) | 4 (40.0) | 0.335 | 3 (42.9) | 4 (57.1) | 7 (70.0) | 3 (30.0) | 0.350 | 5 (71.4) | 2 (28.6) | 7 (70.0) | 3 (30.0) | 1.000 |
| Physician C | 3 (21.4) | 11 (78.6) | 3 (50.0) | 3 (50.0) | 0.303 | 4 (28.6) | 10 (71.4) | 3 (50.0) | 3 (50.0) | 0.613 | 4 (28.6) | 10 (71.4) | 4 (66.7) | 2 (33.3) | 0.161 |
| Physician D | 4 (36.4) | 7 (63.6) | 5 (41.7) | 7 (58.3) | 1.000 | 4 (36.4) | 7 (63.6) | 9 (75.0) | 3 (25.0) | 0.100 | 4 (36.4) | 7 (63.6) | 9 (75.0) | 3 (25.0) | 0.100 |
| Physician E | 4 (44.4) | 5 (55.6) | 12 (85.7) | 2 (14.3) | 0.066 | 5 (55.6) | 4 (44.4) | 12 (85.7) | 2 (14.3) | 0.162 | 5 (55.6) | 4 (44.4) | 12 (85.7) | 2 (14.3) | 0.162 |
| Physician F | 5 (38.5) | 8 (61.5) | 4 (33.3) | 8 (66.7) | 1.000 | 6 (46.2) | 7 (53.8) | 6 (50.0) | 6 (50.0) | 1.000 | 6 (46.2) | 7 (53.8) | 7 (58.3) | 5 (41.7) | 0.695 |
| Baseline knowledge score |  |  |  |  |  |  |  |  |  |  |  |  |  |  |  |
| <60 | 12 (27.9) | 31 (72.1) | 23 (60.5) | 15 (39.5) | 0.004 | 16 (37.2) | 27 (62.8) | 25 (65.8) | 13 (34.2) | 0.014 | 17 (39.5) | 26 (60.5) | 27 (71.1) | 11 (28.9) | 0.007 |
| ≥60 | 10 (34.5) | 19 (65.5) | 20 (62.5) | 12 (37.5) | 0.041 | 13 (44.8) | 16 (55.2) | 24 (75.0) | 8 (25.0) | 0.020 | 14 (48.3) | 15 (51.7) | 25 (78.1) | 7 (21.9) | 0.019 |
| Difference in knowledge scores |  |  |  |  |  |  |  |  |  |  |  |  |  |  |  |
| ≦10 | 16 (32.6) | 33 (67.4) | 17 (54.8) | 14 (45.2) | 0.064 | 22 (44.9) | 27 (55.1) | 20 (64.5) | 11 (35.5) | 0.110 | 23 (46.9) | 26 (53.1) | 20 (64.5) | 11 (35.5) | 0.168 |
| >10 | 6 (26.1) | 17 (73.9) | 26 (66.7) | 13 (33.3) | 0.003 | 7 (30.4) | 16 (69.6) | 29 (74.4) | 10 (25.6) | 0.001 | 8 (34.8) | 15 (65.2) | 32 (82.1) | 7 (17.9) | <0.001 |
